# Epidemiology and health care utilization of patients suffering from Huntington’s disease in Germany: real world evidence based on German claims data

**DOI:** 10.1186/s12883-019-1556-3

**Published:** 2019-12-10

**Authors:** Christoph Ohlmeier, Kai-Uwe Saum, Wolfgang Galetzka, Dominik Beier, Holger Gothe

**Affiliations:** 1 IGES Institut GmbH, Friedrichstraße 180, 10117 Berlin, Germany; 2 Roche Pharma AG, Grenzach-Wyhlen, Germany; 3 InGef - Institute for Applied Health Research Berlin GmbH, Berlin, Germany; 4 Department of Health Sciences / Public Health, Med. Faculty, TU Dresden, Dresden, Germany; 5 Department of Public Health, Health Services Research and HTA, UMIT, Hall in Tirol, Austria

**Keywords:** Huntington’s disease, Claims data analysis, Health care utilization, Epidemiology, Germany, Prevalence, Incidence

## Abstract

**Background:**

Huntington’s disease (HD) is a rare, genetic, neurodegenerative and ultimately fatal disease with no cure or progression-delaying treatment currently available. HD is characterized by a triad of cognitive, behavioural and motor symptoms. Evidence on epidemiology and management of HD is limited, especially for Germany. This study aims to estimate the incidence and prevalence of HD and analyze the current routine care based on German claims data.

**Methods:**

The source of data was a sample of the Institute for Applied Health Research Berlin (InGef) Research Database, comprising data of approximately four million insured persons from approximately 70 German statutory health insurances. The study was conducted in a retrospective cross-sectional design using 2015 and 2016 as a two-year observation period. At least two outpatient or inpatient ICD-10 codes for HD (ICD-10: G10) during the study period were required for case identification. Patients were considered incident if no HD diagnoses in the 4 years prior to the year of case identification were documented. Information on outpatient drug dispensations, medical aids and remedies were considered to describe the current treatment situation of HD patients.

**Results:**

A 2-year incidence of 1.8 per 100,000 persons (95%-Confidence interval (CI): 1.4–2.4) and a 2-year period prevalence of 9.3 per 100,000 persons (95%-CI: 8.3–10.4) was observed. The prevalence of HD increased with advancing age, peaking at 60–69 years (16.8 per 100,000 persons; 95%-CI: 13.4–21.0) and decreasing afterwards.

The most frequently observed comorbidities and disease-associated symptoms in HD patients were depression (42.9%), dementia (37.7%), urinary incontinence (32.5%), extrapyramidal and movement disorders (30.5%), dysphagia (28.6%) and disorders of the lipoprotein metabolism (28.2%).

The most common medications in HD patients were antipsychotics (66.9%), followed by antidepressants (45.1%). Anticonvulsants (16.6%), opioids (14.6%) and hypnotics (9.7%) were observed less frequently.

Physical therapy was the most often used medical aid in HD patients (46.4%). Nursing services and speech therapy were used by 27.9 and 22.7% of HD patients, respectively, whereas use of psychotherapy was rare (3.2%).

**Conclusions:**

Based on a representative sample, this study provides new insights into the epidemiology and routine care of HD patients in Germany, and thus, may serve as a starting point for further research.

## Background

Huntington’s disease (HD) is a rare, genetic, neurodegenerative and ultimately fatal disease with no cure or progression-delaying treatment currently available. HD is caused by a trinucleotide (cytosine-adenine-guanine [CAG]) repeat expansion in the huntingtin gene [1–3]. Typically, HD patients present with a triad of cognitive, behavioral and motor symptoms [3–5], which lead to increasing disability, functional decline and loss of independence [2, 5]. This results in complex patient needs, which are best addressed with interdisciplinary teams consisting of general practitioners, specialists and other health care professionals, e. g. nurses, physical and speech therapists or dietitians [4].

With an estimated prevalence of 3.6 to 5.7 per 100,000 in regions mainly comprising residents of Caucasian descent [6, 7], HD is considered a rare disease in Europe [8]. Although ‘gene silencing’ could be a potential therapeutic strategy in the future [9], HD is currently incurable, and therapeutic approaches focus on symptom management and maintaining the quality of life.

In Germany, information about the epidemiology and management of HD is scarce. German studies are restricted to specific regions and are outdated [10, 11], limiting the generalizability and validity of these studies to current healthcare practice. Furthermore, international studies are only partially transferable to the German healthcare system. Current, real-world data could be used to improve healthcare planning in order to better meet the needs of affected patients [12]. Therefore, the aim of the present study was to fill this evidence gap for Germany by (i) estimating the incidence and period prevalence of HD, (ii) characterizing the demographics and comorbidities of HD patients and (iii) describing the current routine treatments and associated costs.

## Methods

### Data source

The data source for the present study was the Institute for Applied Health Research Berlin (InGef) Research Database. The InGef Research Database is an anonymized claims database with approximately 70 German statutory health insurances (SHI) contributing longitudinal data from approximately 6.7 million persons. For this analysis, the InGef Research Database was condensed to a sample of approximately 4 million persons considered representative of the German population in terms of age and sex, and showing high external validity regarding morbidity, mortality and prescription drug use [13]. This database also contains information on hospitalizations, outpatient physician visits and outpatient drug prescriptions. The hospital data comprises information on the date of admission and discharge, the reason for discharge, diagnostic and therapeutic procedures with the exact date as well as diagnoses, which can be distinguished in hospital main discharge diagnoses and secondary diagnoses. The outpatient data also comprises information on diagnostic and therapeutic procedures with their exact date. Outpatient diagnoses can be distinguished by confirmed diagnoses, suspected diagnoses, status post diagnoses and diagnoses ruled out. Inpatient and outpatient diagnoses are coded according to the German Modification of the International Classification of Diseases 10th Revision (ICD-10-GM). Data on outpatient prescriptions of reimbursed drugs comprise information on the prescription, the date of prescription and the pharmaceutical reference number. The anatomical-therapeutic-chemical code (ATC-code), the defined daily dose (DDD), the packaging size as well as the strength and formulation of the drug can be linked for each dispensed drug based on a pharmaceutical reference database [13].

### Study design

To estimate the prevalence and incidence of HD, a retrospective, cross-sectional study was carried out. This study covered an observation period of 24 months (1 January 2015 to 31 December 2016). This study was descriptive; thus, no hypotheses were pre-specified.

A 24-month observation period was chosen instead of e. g. a 12-month observation period in order to be able to identify a sufficiently large number of HD cases and thus ensure a comparatively robust estimation of the HD burden. Furthermore, if case numbers had been too small, data protection reasons would not have allowed a publication of respective results and would therefore lead to less meaningful results.

### Study population

Insured persons were included if they had continuous insurance coverage during the entire study period or until death during the study period. Analyses on characteristics and prescribed medication were carried out in the subpopulation of patients fulfilling the identification criteria for HD.

### Identification of patients with Huntington’s disease

Patients were identified as prevalent HD cases if at least two diagnoses of HD (ICD-10-GM: G10) were recorded during the study period. Hospital main discharge diagnoses, hospital secondary discharge diagnoses and confirmed outpatient diagnoses were considered for HD case identification. An incident HD case was assumed if patients additionally had no documented hospital main discharge diagnoses, hospital secondary discharge diagnoses or confirmed outpatient diagnoses in the four calendar years prior to the year of case identification.

### Ascertainment of comorbidities and disease-associated symptoms

The burden of specific comorbidities and disease-associated symptoms was ascertained by considering confirmed outpatient diagnoses, hospital main discharge diagnoses and hospital secondary diagnoses documented during the 24-months observation period. One diagnosis was sufficient to be identified as having the comorbidity or disease-associated symptom (see Additional file 3). A data-driven approach of the most frequently documented diseases was carried out based on the three-digit level of the ICD-10-GM code (see Additional file 4).

### Ascertainment of prescribed drugs, medical aids and remedies

All analyses regarding HD treatment were based on the 24-month observation period. Outpatient drugs were classified by three- or four-digit level of the ATC-code; specific drug were classified by seven-digit level of the ATC-code. One dispensation of a drug or drug class was sufficient to meet the criteria. Furthermore, the sum of dispensed DDD and the accumulated costs (pharmacy retail price) during the 24-month period were ascertained. In addition, the most frequently dispensed drugs were analyzed based on the seven-digit level of the ATC-code.

At least one claim of medical aids and remedies of a respective service was sufficient to meet the criteria. Accumulated costs to the SHI during the observation period were calculated for each respective care service.

### Statistical analyses

The two-year incidence and the two-year period prevalence of HD were calculated by dividing the number of incident and prevalent HD cases, respectively, by the study population. Both estimates are presented per 100,000 persons.

The proportion of patients with a specific comorbidity or disease-associated symptom; use of a specific drug or class; or use of medical aid or remedy were calculated by dividing the number of patients fulfilling the respective criteria by the number of persons in the HD population.

Mean costs of health service was calculated by dividing the sum of costs by the number of patients receiving the respective health service. To ascertain the mean costs per year, the mean costs were divided by two, assuming that the mean resource use was equally distributed in both calendar years. The analyses of the costs of HD treatment are therefore purely descriptive. A more comprehensive analysis of the economic disease burden was not the subject of this study.

All analyses were carried out stratified by sex.

All analyses were carried out using R, version 3.5.0.

## Results

### Patient characteristics

Of the 308 patients identified with HD, 45.1% were female (Table 1). The mean age of prevalent HD patients was 59.8 years (standard deviation (SD): 13.6) with the majority being between 50 and 69 years old.

**Table 1:** Characteristics of patients with prevalent HD in 2015 and 2016

	Women (*n* = 139)		Men (*n* = 169)		All (*n* = 308)	
	n	%	n	%	n	%
Age						
0–29 years	< 5	/	/	/	6	1.9%
30–39 years	5	3.6%	10	5.9%	15	4.9%
40–49 years	18	12.9%	29	17.2%	47	15.3%
50–59 years	38	27.3%	48	28.4%	86	27.9%
60–69 years	42	30.2%	35	20.7%	77	25.0%
70–79 years	24	17.3%	34	20.1%	58	18.8%
> =80 years	9	6.5%	10	5.9%	19	6.2%
All	139	100.0%	169	100.0%	308	100.0%
Mean age (mean, SD)	60.4	13.3	59.4	13.4	59.8	13.6
Sex						
Female	139	100.0%	–	–	139	45.1%
Male	–	–	169	100.0%	169	54.9%

Numbers which are too low or might allow indirect calculability of too low case numbers cannot be displayed due to data protection reasons

*SD* Standard deviation

The most common comorbidity or disease-associated symptom was depression (42.9%), which was slightly more frequent in women than in men (Table 2). Extrapyramidal and movement disorders were diagnosed in 30.5% of HD patients, and dysphagia was diagnosed in 28.6% of HD patients. Additionally, the data-driven analysis showed that more than one third of the HD patients were affected by dementia, approximately 30% had urinary incontinence, and approximately 30% had disorders of the lipoprotein metabolism. Female HD patients were more frequently affected by lipoprotein disorders than males (see Additional file 2).

**Table 2:** Pre-specified comorbidities and disease-associated symptoms of patients with prevalent HD in 2015 and 2016

	Women (*n* = 139)		Men (*n* = 169)		All (*n* = 308)	
	n	%	n	%	n	%
ADHD	8	5.8%	8	4.7%	16	5.2%
Anxiety	26	18.7%	12	7.1%	38	12.3%
Bipolar disorder	< 5	/	/	/	< 5	/
Depression	64	46.0%	68	40.2%	132	42.9%
Diabetes mellitus	16	11.5%	18	10.7%	34	11.0%
Dysphagia	35	25.2%	53	31.4%	88	28.6%
Extrapyramidal and movement disorders	42	30.2%	52	30.8%	94	30.5%
Insomnia	20	14.4%	21	12.4%	41	13.3%
Obsessive compulsive disorder	< 5	/	/	/	< 5	/
Osteoarthritis	31	22.3%	24	14.2%	55	17.9%
Other systemic atrophies (excl. HD)	< 5	/	/	/	8	2.6%

Numbers which are too low or might allow indirect calculability of too low case numbers cannot be displayed due to data protection reasons

### Incidence and prevalence of HD

The overall two-year incidence of HD was 1.8 per 100,000 persons (95%-Confidence interval (CI): 1.4–2.4). The overall two-year prevalence of HD (9.3 per 100,000; 95%-CI: 8.3–10.4) was higher in men (10.2 per 100,000; 95%-CI: 8.8–11.9) than in women (8.3 per 100,000; 95%-CI: 7.0–9.8). The prevalence of HD increased with advancing age, peaking at 60–69 years (16.8 per 100,000 persons; 95%-CI: 13.4–21.0) and declining in patients aged 70 years or older (Table 3).

**Table 3:** Age- and sex-stratified two-year prevalence of HD

	Women (*n* = 139)			95%-CI	Men (*n* = 169)			95%-CI	All (*n* = 308)			95%-CI
	N population	n cases	Prevalence per 100,000 persons		N population	n cases	Prevalence per 100,000 persons		N population	n cases	Prevalence per 100,000 persons	
Age												
0–29 years	426,131	< 5	–		440,649	/	–		866,780	6	0.7	0.3–1.5
30–39 years	173,714	5	2.9	1.2–6.7	172,856	10	5.8	3.1–10.6	346,570	15	4.3	2.6–7.1
40–49 years	221,720	18	8.1	5.1–12.8	223,105	29	13.0	9.1–18.7	444,825	47	10.6	7.9–14.0
50–59 years	284,651	38	13.3	9.7–18.3	289,813	48	16.6	12.5–22.0	574,464	86	15.0	12.1–18.5
60–69 years	235,666	42	17.8	13.2–24.1	222,871	35	15.7	11.3–21.8	458,537	77	16.8	13.4–21.0
70–79 years	193,336	24	12.4	8.3–18.5	192,799	34	17.6	12.6–24.6	386,135	58	15.0	11.6–19.4
> =80 years	141,035	9	6.4	3.4–12.1	107,292	10	9.3	5.1–17.2	248,327	19	7.7	4.9–12.0
All	1,676,253	139	8.3	7.0–9.8	1,649,385	169	10.2	8.8–11.9	3,325,638	308	9.3	8.3–10.4

*CI* Confidence interval

### Use of drugs, medical aids and remedies

Antipsychotic drugs were the most frequently observed dispensed drugs in prevalent HD patients with 66.9% receiving at least one dispensation of antipsychotics, mean dispensed DDD per year of 386.5 and associated mean costs of €556.64 per year (Table 4). Tiapride was the most frequently dispensed antipsychotic drug (46.8% of HD patients) (see Additional file 1). Furthermore, 45.1% of the HD patients received antidepressants with mean costs of €125.22 per year and with mean DDD of 352.3 per year. The most frequently observed antidepressant drug was mirtazapine with 20.5% of HD patients with at least one dispensation (see Additional file 1). Use of anticonvulsants, hypnotics and opioids was observed in substantially fewer patients. Sex-specific differences regarding drug use were rarely observed. However, mean annual DDD of antipsychotic drugs were higher in women compared to men. Besides tiapride and mirtazapine metamizole (29.5%), ibuprofen (25.3%) and pantoprazole (24.4%) were the most frequently dispensed drugs in HD patients. Tetrabenazine, which is approved for the treatment of hyperkinetic movement disorders in HD, was dispensed to 18.5% of HD patients.

**Table 4:** Use of drugs, medical aids and remedies of prevalent HD patients in 2015 and 2016

	Women (*n* = 139)				Men (*n* = 169)				All (*n* = 308)			
	n	%	Mean costs per patient and year	Mean DDD per year	n	%	Mean costs per patient and year	Mean DDD per year	n	%	Mean costs per patient and year	Mean DDD per year
Drugs												
Anticonvulsants	21	15.1%	153.76 €	132.9	30	17.8%	146.93 €	124.7	51	16.6%	149.74 €	128.1
Antidepressants	62	44.6%	129.50 €	356.4	77	45.6%	121.78 €	349.1	139	45.1%	125.22 €	352.3
Antipsychotics	91	65.5%	633.34 €	467.6	115	68.0%	495.95 €	322.2	206	66.9%	556.64 €	386.5
Hypnotics	15	10.8%	67.54 €	86.6	15	8.9%	43.22 €	84.0	30	9.7%	55.38 €	85.3
Opioids	20	14.4%	214.81 €	47.6	25	14.8%	90.98 €	33.7	45	14.6%	146.02 €	39.9
Medical aids												
Nursing^a^	40	28.8%	–	–	46	27.2%	–	–	86	27.9%	–	–
Physical therapy	65	46.8%	795.43 €	–	78	46.2%	791.01 €	–	143	46.4%	793.02 €	–
Psychotherapy	/	/	/	–	< 5	/	/	–	10	3.2%	553.39 €	–
Speech therapy	28	20.1%	1232.98 €	–	42	24.9%	966.42 €	–	70	22.7%	1073.04 €	–
Remedies												
Walking aid	14	10.1%	128.33 €	–	15	8.9%	61.28 €	–	29	9.4%	93.65 €	–
Wheelchair use	27	19.4%	481.91 €	–	29	17.2%	591.71 €	–	56	18.2%	538.77 €	–

Numbers which are too low or might allow indirect calculability of too low case numbers cannot be displayed due to data protection reasons

^a^Costs for nursing services were not comprised by the database

Physical therapy was the most frequent medical aid with 46.4% of HD patients receiving at least one prescription. Physical therapy was associated with the mean annual costs of €793.02. Speech therapy was prescribed to 22.7% of HD patients with mean annual costs of €1073.04 per year. In addition, 18.2% of patients with HD used a wheel chair and 9.4% a walking aid. Sex-specific differences regarding use of medical aids and remedies were rarely observed.

## Discussion

### Summary of findings

Based on representative German claims data, a two-year incidence for HD of 1.8 per 100,000 persons and a two-year prevalence of 9.3 per 100,000 persons was observed. This study also showed that depression was the most frequent comorbidity or disease-associated symptom in HD patients. Furthermore, two thirds of HD patients received antipsychotics during the observation period.

### Discussion of findings

Several systematic reviews on the prevalence of HD have been published, which all point towards a heterogeneous body of evidence [6, 7, 14]. According to Pringsheim et al., pooled evidence from North America, Europe and Australia resulted in a prevalence of 5.70 per 100,000 (95% CI: 4.42–7.35) which ranged from 1.56 to 12.08 per 100,000 persons [6]. Rawlins et al. reported a prevalence of 3.60 (95%-CI: 3.50–3.69) for Western Europe which ranged from 0.53 to 10.85 per 100,000 persons [7]. Baig et al. presented heterogeneous study results with a prevalence ranging from 0.96 to 13.7 per 100,000 persons for North America, Europe and Australia [14]. Our estimate, at 9.3 per 100,000 persons, is higher than reported in most systematic reviews. This could be due to several reasons. Firstly, the studies in the reviews – including those from German population samples – are mostly older than 20 years [10, 11]. Evidence suggests that the prevalence of HD has increased during the past 30 years [7, 15], because of higher awareness among physicians [7], longer survival of the patients [16] and progression of the baby-boomers’ generation into the manifest phase of HD [17]. Thus, a higher prevalence in our study was to be expected. Secondly, there seems to be a considerably higher HD prevalence in Caucasian populations compared to non-Caucasian populations [7, 18, 19]. The higher prevalence in the present study compared to the reviews could be explained by comparatively high proportion of residents with Caucasian descent in Germany. A recent study in the United Kingdom (UK) observed a prevalence of 12.3 per 100,000 persons for patients older than 20 years [15], a prevalence comparable to that of our study. Evans et al., whose research is based on the General Practice Research Database (GPRD; now Clinical Practice Research Datalink (CPRD)) comprising anonymized medical records from the primary care setting, analyzed data between 1990 and 2010. During this period, the database grew from approximately 650,000 to more than 3,500,000 patient records, allowing them to determine the prevalence in narrow age categories and in specific regions. Similar to our results, Evans et al. found only slight differences in the average prevalence for women (10.4 per 100,000 persons) and men (9.4 per 100,000 persons). However, they believe their estimates to be too low due to undetected cases of this rare condition [15]. Regarding the higher prevalence in older age groups, our findings generally align with others [20]. Nevertheless, in other studies, the average prevalence peaks between 51 and 60 years [15] and 60–64 years [20], whereas we found the highest prevalence between 60 and 69 years.

In addition, several studies have determined the incidence of HD. In their systematic review, Pringsheim and colleagues reveal a mean incidence of 0.4 per 100,000 persons per year with a higher incidence in studies including non-Asian populations (0.1–0.8 per 100,000 persons) than in studies with populations of Asian descent (0.05–0.1 per 100,000 persons) [6]. A study from Italy, which was based on administrative data and medical records, estimated the incidence of HD to be 0.3 per 100,000 persons [21]. In another study based on CPRD data, an incidence of 0.7 per 100,000 persons was observed with no trend in the incidence of HD over time (1990–2010) [22]. The incidence of HD determined in our study is comparatively high (1.8 per 100,000 persons). The difference, however, is comparatively small considering a two-year observation period chosen in our study.

Depression is a common comorbidity / disease-associated symptom of HD discussed in the literature. Our results of the depression rate within the HD patient population were consistent with previous research [23, 24], although we could not distinguish between HD stages due to the type of our underlying dataset. A comparison of our data with self-reported depression and anxiety data in a Norwegian cohort using the EQ-5D-3 L instrument [25] is not valid because evidence from studies that screen validated questionnaires point to an underestimation of the frequency of depression in claims data [26]. However, at the same time, German claims-data based studies revealed higher estimations of the prevalence of depression compared to national survey data [27]. According to the authors, this may be due to a lesser willingness to provide information on sensitive topics in interview situations compared to conversations with a familiar physician [27]. In addition, persons with severe depressions seem to be underrepresented in the study by Frank et al., which also might have contributed to a lower prevalence of depression in survey data compared to claims data [28].

Around 38% of HD patients in our sample had dementia as a comorbidity / disease-associated symptom. However, comparisons with other studies are difficult, as no consistent criteria were applied in these studies. Due to cognitive impairments, which can be present even decades before the diagnosis [29, 30] and could manifest in performance at work, managing finances or safe driving [31], a general definition of dementia in HD might be difficult and criteria for the diagnosis of dementia cannot be applied 1:1 to comorbid dementia in HD patients (for comprehensive discussion see [32, 33]). Since varying definitions of dementia in HD may also affect the coding behavior of physicians, claims-data based analyses of the dementia burden in HD populations must therefore currently be interpreted cautiously.

Anderson et al. [34] studied the healthcare utilization in the United States of America (USA) among Medicaid- and commercial-insured HD patients based on claims data. They report home assistance (which does not equate to nursing in this study) and physical therapy (with a usage rate between 37.1 and 64.0%, depending on condition stage and insurance type) to be the most frequent interventions. Our result of 46.4% of HD patients using physical therapy were within that range. Similarly, the proportion of HD patients in our sample using speech therapy and wheelchairs is within the range reported by Anderson et al. In addition, we report nearly the same percentage of patients using walking aids (around 9.5%) [34].

More than half of the HD patients in the sample were given antipsychotics. However, the prevalence of psychoses in HD patients seems to be considerably lower. A study analyzing factors contributing to psychosis in HD based on the Enroll-HD database, found 10.8% of HD patients with a history of psychosis [35]. Instead, chorea is frequently treated with antipsychotics, especially in Europe [36, 37]. In a survey among HD experts, 50% of European respondents would choose tiapride as first choice therapy for treating chorea [37]. Therefore, the high use of antipsychotics in general and tiapride in particular (46.8% of the sample), hints at the dispension to treat mainly motor deficits. Furthermore, dopamine antagonists like tiapride may show beneficial effects on HD patients regarding mood stabilization, sleep disturbances and the prevention of weight loss which might have also contributed to the comparatively high use of antipsychotics [38, 39].

Although studies on HD about direct and societal costs exist [24, 40], comparability with the present study is limited. This is because of varying research objectives, discrepancies in the displaying of the cost types, and large differences in the organization of healthcare between Germany, the UK and the USA.

### Strengths and limitations

The main strength of this study is the large, unselected and supra-regional sample allowing robust estimations of the epidemiology, comorbidity or disease-associated symptoms and healthcare utilization for HD patients. Furthermore, recall and selection bias, both being a considerable methodological challenge in health services research, could be avoided since this study was based on claims data and did not depend on patient recall or the willingness to participate.

Although we based our findings on representative data, our findings should be interpreted with caution. Due to the cross-sectional design, it was not possible to address changing utilization or trends over time, and we cannot claim a causal relationship of, for example, comorbidity / disease-associated symptoms and drug use. Thus, our study is primarily descriptive. In addition, as we derive our findings from claims data, some limitations regarding this form of information should be considered. First, the nursing sector was not comprised by the database. Respective analyses were nevertheless feasible, since specific outpatient codes indirectly pointed towards use of nursing services. Consequently, the proportion of HD patients with use of nursing services is underestimated and costs could not be analyzed. Informal care, which is a large part of healthcare for the patients, could not be addressed in this study as well. Second, because of a lack of information about the severity of the condition, we were not able to depict the healthcare utilization stratified by stage, which could be of particular interest to healthcare planning. Furthermore, there is a gap between coding a healthcare service and actual utilization; for example, prescription of drugs does not imply full adherence to the medication plan. It should also be considered, that the insidious onset of HD might lead to a delayed diagnosis and therefore to an underestimation of epidemiological estimates.

## Conclusion

To our knowledge, this study is the first comprehensive and supra-regional analysis of epidemiological measures and resource use in patients with HD in Germany. Based on a large and representative sample, this study provides new insights into the characteristics of the patients and their routine care. Thus, this study may serve as a starting point for further research, such as analyses describing treatment pathways and assessing the quality of intersectoral care, which would subsequently allow further development of the care of patients with HD in Germany.

## Supplementary information

**Additional file 1.** Most frequently used drugs (7-digit ATC level) in prevalent HD patients in 2015 and 2016

**Additional file 2.** Most frequently observed comorbidities and disease-associated symptoms (3-digit ICD 10 level) in patients with prevalent HD in 2015 and 2016

**Additional file 3.** Operationalization of pre-specified approach for identification of comorbidities and disease-associated symptoms

**Additional file 4.** Operationalization of data driven approach for identification of comorbidities and disease-associated symptoms

## Data Availability

The authors are not allowed to share the analysis datasets of the current study due to data protection regulations.

## Abbreviations

ATC: Anatomical-therapeutic-chemical
CAG: Cytosine-adenine-guanine
CI: Confidence interval
CPRD: Clinical Practice Research Datalink
DDD: Defined daily dose
GPRD: General Practice Research Database
HD: Huntington’s Disease
ICD-10-GM: German Modification of the International Classification of Diseases 10th Revision
InGef: Institute for Applied Health Research Berlin
SHI: Statutory health insurance
UK: United Kingdom
USA: United States of America
