# Supplementary material for: Epidemiology and health care utilization of patients suffering from Huntington’s disease in Germany: real world evidence based on German claims data
Source: BMC Neurol. 2019 Dec 10;19:318. doi: 10.1186/s12883-019-1556-3 (PMC6905058; doi:10.1186/s12883-019-1556-3)
Supplement: Supplementary file 1 — Additional file 1. Most frequently used drugs (7-digit ATC level) in prevalent HD patients in 2015 and 2016 [file 12883_2019_1556_MOESM1_ESM.docx]

Additional file 1: Most frequently used drugs (7-digit ATC level) in prevalent HD patients in 2015 and 2016

|  | Women (n=139) | | Men (n=169) | | All (n=308) | |
| --- | --- | --- | --- | --- | --- | --- |
|  | n | % | n | % | n | % |
| Tiapride (Antipsychotic) | 64 | 46.0 | 80 | 47.3 | 144 | 46.8 |
| Metamizole (Non-opioid analgesic) | 45 | 32.4 | 46 | 27.2 | 91 | 29.5 |
| Ibuprofen (NSAID) | 38 | 27.3 | 40 | 23.7 | 78 | 25.3 |
| Pantoprazole (Proton pump inhibitor) | 38 | 27.3 | 37 | 21.9 | 75 | 24.4 |
| Mirtazapine (Antidepressant) | 35 | 25.2 | 28 | 16.6 | 63 | 20.5 |
| Lorazepam (Benzodiazepine) | 23 | 16.5 | 38 | 22.5 | 61 | 19.8 |
| Tetrabenazine (Drug for HD treatment) | 19 | 13.7 | 38 | 22.5 | 57 | 18.5 |
| Olanzapine (Antipsychotic) | 15 | 10.8 | 24 | 14.2 | 39 | 12.7 |
| Cefuroxime (β-lactam antibiotic) | 17 | 12.2 | 19 | 11.2 | 36 | 11.7 |
| Risperidone (Antipsychotic) | 12 | 8.6 | 22 | 13.0 | 34 | 11.0 |
