# Supplementary material for: Epidemiology and health care utilization of patients suffering from Huntington’s disease in Germany: real world evidence based on German claims data
Source: BMC Neurol. 2019 Dec 10;19:318. doi: 10.1186/s12883-019-1556-3 (PMC6905058; doi:10.1186/s12883-019-1556-3)
Supplement: Supplementary file 2 — Additional file 2. Most frequently observed comorbidities and disease-associated symptoms (3-digit ICD 10 level) in patients with prevalent HD in 2015 and 2016 [file 12883_2019_1556_MOESM2_ESM.docx]

Additional file 2: Most frequently observed comorbidities and disease-associated symptoms (3-digit ICD 10 level) in patients with prevalent HD in 2015 and 2016

|  | Women (n=139) | | Men (n=169) | | All (n=308) | |
| --- | --- | --- | --- | --- | --- | --- |
|  | n | % | n | % | n | % |
| Depressive episode | 59 | 42.4% | 59 | 34.9% | 118 | 38.3% |
| Dementia | 53 | 38.1% | 63 | 37.3% | 116 | 37.7% |
| Movement disorders | 54 | 38.8% | 58 | 34.3% | 112 | 36.4% |
| Essential hypertension | 42 | 30.2% | 63 | 37.3% | 105 | 34.1% |
| Urinary incontinence | 43 | 30.9% | 57 | 33.7% | 100 | 32.5% |
| Need for immunization | 47 | 33.8% | 43 | 25.4% | 90 | 29.2% |
| Dysphagia | 35 | 25.2% | 53 | 31.4% | 88 | 28.6% |
| Lipoprotein metabolism disorders | 47 | 33.8% | 40 | 23.7% | 87 | 28.2% |
| Problems in the context of long-term care | 39 | 28.1% | 43 | 25.4% | 82 | 26.6% |
| Back pain | 40 | 28.8% | 37 | 21.9% | 77 | 25.0% |
| Dysphasia and aphasia | 31 | 22.3% | 43 | 25.4% | 74 | 24.0% |
| Personality and behavioural disorders | 25 | 18.0% | 46 | 27.2% | 71 | 23.1% |
| Extrapyramidal and movement disorders | 30 | 21.6% | 39 | 23.1% | 69 | 22.4% |
| Faecal incontinence | 23 | 16.5% | 40 | 23.7% | 63 | 20.5% |
| Other functional intestinal disorders | 27 | 19.4% | 34 | 20.1% | 61 | 19.8% |
| Injury of unspecified body region | 29 | 20.9% | 28 | 16.6% | 57 | 18.5% |
| Acute upper respiratory infection | 31 | 22.3% | 21 | 12.4% | 52 | 16.9% |
| Other disorders of external ear | 22 | 15.8% | 28 | 16.6% | 50 | 16.2% |
| Other dermatitis | 27 | 19.4% | 22 | 13.0% | 49 | 15.9% |
| Disorders of refraction and accommodation | 28 | 20.1% | 20 | 11.8% | 48 | 15.6% |
| Numbers which are too low or might allow indirect calculability of too low case numbers cannot be displayed due to data protection reasons. | | | | | | |
