# Supplementary material for: Epidemiology and health care utilization of patients suffering from Huntington’s disease in Germany: real world evidence based on German claims data
Source: BMC Neurol. 2019 Dec 10;19:318. doi: 10.1186/s12883-019-1556-3 (PMC6905058; doi:10.1186/s12883-019-1556-3)
Supplement: Supplementary file 3 — Additional file 3. Operationalization of pre-specified approach for identification of comorbidities and disease-associated symptoms [file 12883_2019_1556_MOESM3_ESM.docx]

Additional file 3: Operationalization of pre-specified approach for identification of comorbidities and disease-associated symptoms

|  | ICD 10 GM Code |
| --- | --- |
| ADHD | F90 |
| Anxiety | F40, F41 |
| Bipolar disorder | F31 |
| Depression | F32, F33 |
| Diabetes mellitus | E10-E14 |
| Dysphagia | R13 |
| Extrapyramidal and movement disorders | G20-G26 |
| Insomnia | G47 |
| Obsessive compulsive disorder | F42 |
| Osteoarthritis | M15-M19 |
| Other systemic atrophies (excl. HD) | G10-G14 |
| GM: German Modification | |
