# Supplementary material for: Epidemiology and health care utilization of patients suffering from Huntington’s disease in Germany: real world evidence based on German claims data
Source: BMC Neurol. 2019 Dec 10;19:318. doi: 10.1186/s12883-019-1556-3 (PMC6905058; doi:10.1186/s12883-019-1556-3)
Supplement: Supplementary file 4 — Additional file 4. Operationalization of data driven approach for identification of comorbidities and disease-associated symptoms [file 12883_2019_1556_MOESM4_ESM.docx]

Additional file 4: Operationalization of pre-specified approach for identification of comorbidities and disease-associated symptoms

|  | ICD 10 GM Code |
| --- | --- |
| Depressive episode | F32 |
| Dementia | F02 |
| Movement disorders | R26 |
| Essential hypertension | I10 |
| Urinary incontinence | R32 |
| Need for immunization | Z25 |
| Dysphagia | R13 |
| Lipoprotein metabolism disorders | E78 |
| Problems in the context of long-term care | Z74 |
| Back pain | M54 |
| Speech disturbances | R47 |
| Personality and behavioural disorders | F07 |
| Extrapyramidal and movement disorders | G25 |
| Faecal incontinence | R15 |
| Other functional intestinal disorders | K59 |
| Injury of unspecified body region | T14 |
| Acute upper respiratory infection | J06 |
| Other disorders of external ear | H61 |
| Other dermatitis | L30 |
| Disorders of refraction and accommodation | H52 |
| GM: German Modification | |
